# Auditory Phenotypic Variability in Friedreich’s Ataxia Patients

**DOI:** 10.1007/s12311-021-01236-9

**Published:** 2021-02-18

**Authors:** Nehzat Koohi, Gilbert Thomas-Black, Paola Giunti, Doris-Eva Bamiou

**Affiliations:** 1 The Ear Institute, University College London, London, WC1X 8EE UK; 2 Neuro-otology Department, University College London Hospitals, London, WC1E 6DG UK; 3 Department of Clinical and Movement Neurosciences, Institute of Neurology, University College London, London, WC1N 3BG UK; 4 Ataxia Centre, National Hospital for Neurology and Neurosurgery, University College London Hospitals, London, WC1N 3BG UK; 5 Biomedical Research Centre, National Institute for Health Research, London, WC1E 6DG UK

**Keywords:** Friedreich’s ataxia,, Cognition,, Hearing,, Auditory processing,, Phenotype,, GAA1

## Abstract

Auditory neural impairment is a key clinical feature of Friedreich’s Ataxia (FRDA). We aimed to characterize the phenotypical spectrum of the auditory impairment in FRDA in order to facilitate early identification and timely management of auditory impairment in FRDA patients and to explore the relationship between the severity of auditory impairment with genetic variables (the expansion size of GAA trinucleotide repeats, GAA1 and GAA2), when controlled for variables such as disease duration, severity of the disease and cognitive status. Twenty-seven patients with genetically confirmed FRDA underwent baseline audiological assessment (pure-tone audiometry, otoacoustic emissions, auditory brainstem response). Twenty of these patients had additional psychophysical auditory processing evaluation including an auditory temporal processing test (gaps in noise test) and a binaural speech perception test that assesses spatial processing (Listening in Spatialized Noise-Sentences Test). Auditory spatial and auditory temporal processing ability were significantly associated with the repeat length of GAA1. Patients with GAA1 greater than 500 repeats had more severe auditory temporal and spatial processing deficits, leading to poorer speech perception. Furthermore, the spatial processing ability was strongly correlated with the Montreal Cognitive Assessment (MoCA) score. To our knowledge, this is the first study to demonstrate an association between genotype and auditory spatial processing phenotype in patients with FRDA. Auditory temporal processing, neural sound conduction, spatial processing and speech perception were more severely affected in patients with GAA1 greater than 500 repeats. The results of our study may indicate that auditory deprivation plays a role in the development of mild cognitive impairment in FRDA patients.

## Introduction

Friedreich’s Ataxia (FRDA) is the most frequent autosomal recessive inherited ataxia caused by mutations in the *FXN* gene. An expansion of a homozygous or heterozygous GAA trinucleotide repeat in intron 1 of the gene, which results in deficiency of the mitochondrial protein frataxin, is present in more than 96% of mutant alleles [1]. Cardinal features of FRDA are ataxia of both trunk and limbs along with dysarthria, global areflexia, deep sensory loss and pyramidal signs [1]. Sensorineural hearing loss as revealed in a pure-tone audiogram is seen in only 8 to 13% of FRDA patients but other types of hearing impairment, such as difficulty understanding speech in background noise, are present in more than 90% of these patients [1–3]. Preneural auditory responses from the cochlear outer hair cells are typically normal in more than 90% of the patients [4]. Most of these individuals show abnormalities in auditory neural and brainstem responses as a result of axonopathy in the eighth nerve and auditory brainstem, termed as “auditory neuropathy,” [4–6] which is the cause of listening difficulties [4, 7, 8]. Patients with auditory neuropathy struggle to understand speech when background noise is present [9, 10] and show impaired ability to selectively attend to a particular voice based on its location [7].

There is phenotypic variability in FRDA that may, to some extent, be explained by the molecular mechanism underlying the disease. The expansion size of the number of GAA trinucleotide repeats on the shorter allele (hereafter referred to as GAA1) in each pair inversely correlates with age at onset and directly with the presence of diabetes mellitus, cardiomyopathy and severity of peripheral sensory neuropathy [1, 5, 11–13]. In this connection, the size of GAA1 is more significant than the size of the larger allele (GAA2). There is also auditory phenotypic variability (see Table 1 for a summary of studies) but only a few studies attempted to correlate the size of GAA1 with the severity of auditory impairment. Apart from one study conducted by Rance et al. [4], all showed no correlation between the size of GAA1 and the severity of auditory impairment (Table 1). Rance’s study is the single study [4] that reported a significant correlation between an auditory temporal processing test (of amplitude modulation detection, i.e. the ability to detect changes in frequency) and GAA1. Other studies reported no correlation between the repeat length of GAA1 and the severity of auditory processing impairment as reflected by neurophysiology (auditory brainstem evoked responses) or other psychoacoustic tests including speech in noise. As FRDA is a rare disease, these studies had small samples and almost all employed a fairly limited set of tests in order to assess the auditory impairment in FRDA in detail and its correlation with the genotype. Identifying and understanding such associations would be of benefit in the search of biomarkers for this disease.

**Table 1:** Audiological abnormalities in FRDA individuals

Study	Audiological tests	Hearing impairment	GAA correlation performed?	No of patients	Age range	GAA range
Jabbari et al., 1983 [5]	ABR, ART, Tymp	100% abnormal ABR	No	5		-
Durr et al., 1996 [1]	PTA, ABR	13% abnormal PTA, 61% abnormal ABR	Yes (no correlation was found)	140 (only 69 had ABR)	7–77	120–1700
Santoro et al., 2000 [14]	ABR	-	Yes (no correlation was found)	24	9–43	200–1093
Rance et al., 2008 [10]	PTA, ABR, ART, speech in noise test	30% abnormal ABR and ART, 90% abnormal speech in noise	Yes, (no correlation was found)	10	8–28	447–780
Rance et al., 2010 [4]	PTA, OAEs, ABR, temporal processing test	64% with temporal processing deficit, 50% abnormal ABR,	Yes, (significant correlation was found only between amplitude modulation detection and GAA1)	14	16–52	447–1099
Rance et al., 2012 [8]	PTA, ABR, LiSN-S	52% abnormal ABR, 22% abnormal PTA (4 freq. average)	Yes (no correlation was found between LiSN-S subscores and GAA1)	23	9–55	447–1298
Zeigelboim et al., 2018 [15]	PTA, ABR, Immittance	43% abnormal PTA, 57% abnormal ABR, 50% abnormal immittance	No	30	6–72	-
Giroudet et al., 2018 [16]	PTA, OAEs, standard ABR, split ABR, speech in noise	24% abnormal PTA, 75% abnormal speech in noise, 92% abnormal standard ABR, 38% abnormal split ABR	No	37	12–63	-
Koohi et al., (present study)	PTA, OAEs, ABR, SiQ, SiB, LiSN-S, GIN	45% abnormal PTA, 15% abnormal OAEs, 77% abnormal ABR, 77% abnormal SiQ, 100% abnormal SiB, 95% abnormal LiSN-S, 77% abnormal GIN	Yes	27	17–58	100–1050

ABR, auditory brainstem response; PTA, pure-tone audiometry; Tymp, tympanometry; ART, acoustic reflex threshold; OAEs, otoacoustic emissions; GIN, gaps in noise; LiSN-S, Listening in Spatialized; Noise Sentences Test SiB, speech in babble test; SiQ, speech in quiet

Our main aim was to characterize the phenotypical spectrum of the auditory sensitivity and auditory processing impairment in FRDA in more detail in order to explore further if the auditory phenotypes correlate with genetic and clinical variables such as GAA1, GAA2, disease duration, severity of the disease and cognitive status. Our exploratory aim was to measure the relationship between patients’ auditory processing and cognitive status as one might affect the other [17, 18].

## Methods

### Standard Protocol Approvals, Registrations and Patient Consents

This study was registered and conducted as an audit at the University College London Hospitals (UCLH). All procedures were conducted as part of routine clinical care. The study was performed under the ethical guidelines issued by our institution, with written informed consent obtained from all participants.

### Participants and Settings

Our neuro-otology clinic is part of The Ataxia Centre’s multidisciplinary team at UCLH. Between 2016 and 2020, clinicians working in the Ataxia Centre referred twenty-seven FRDA patients, who carried GAA expansions in intron 1 of *FXN* gene, to the Neuro-otology Department of UCLH for hearing assessment. From this sample, all patients had pure-tone audiometry and otoacoustic emissions; 26 patients had auditory brainstem response; 21 speech in quiet test; 18 speech in babble test; 20 listening in spatialized noise test; and 21 gaps in noise test. Diagnostic details for each individual with FRDA were collected (Table 2). Age at assessment ranged from 17 to 58 years (32.8 ± 11.4 years). The number of GAA1 and GAA2 repeats ranged from 100 to 1050 (684 ± 258) and 220 to 1680 (948 ± 261), respectively. The disease duration, which was calculated by subtracting the age at onset from the age of the patient at assessment, ranged from 7 to 44 years (22.6 ± 10.5). Patients were classified into three groups, those with GAA1 repeats over 700, those between 500 and 700 and those with repeats under 500 [1]. No significant statistical differences were found in disease duration, age at assessment, age at onset and Scale for the Assessment and Rating of Ataxia (SARA) scores between the three GAA groups.

**Table 2:** FRDA patient details

Subject	Age at assessment (years), gender	Age at onset (years)	Disease duration (years)	GAA1	GAA2	SARA
FRDA1*	18, F	11	7	100	650	12
FRDA2*	36, F	2	34	150	850	26
FRDA3*	17, M	4	13	220	220	27.5
FRDA4*	48, F	17	31	400	834	30
FRDA5*	42, F	17	25	400	800	23
FRDA6*	45, F	15	30	467	967	26
FRDA7**	37, F	19	28	567	900	30
FRDA8**	40, M	20	20	567	967	20
FRDA9**	21, M	6	15	580	745	23
FRDA10**	30, M	11	19	634	767	30
FRDA11**	58, F	15	43	683	983	40
FRDA12**	18, M	5	15	700	850	16
FRDA13***	40, F	8	32	720	920	39
FRDA14***	28, F	5	23	745	945	29
FRDA15***	34, M	8	26	785	785	30
FRDA16***	26, M	8	18	800	867	26.6
FRDA17***	51, F	7	44	820	820	37
FRDA18***	24, F	12	12	834	1034	17
FRDA19***	41, M	7	34	834	1167	38
FRDA20***	20, M	8	12	850	850	20.5
FRDA21***	22, M	15	7	850	1200	21.5
FRDA22***	30, M	11	19	850	1350	36
FRDA23***	50, M	13	37	850	1050	28
FRDA24***	31, F	22	9	1000	1000	21
FRDA25***	25, F	7	18	1000	1200	40
FRDA26***	31, F	3	28	1020	1220	40
FRDA27***	21, M	5	16	1050	1680	29

*, GAA1 < 500; **, GAA1 500–700; ***GAA1 > 700

### Baseline Audiological Assessment

We collected information about the patients’ hearing status. After otoscopy and wax removal, patients were tested in a sound-treated booth with the following test procedures as per the standard protocol of the clinic and in accordance with test recommendations by the British Society of Audiology [19].

- Pure-tone audiometry (PTA)—PTA was tested at frequencies 250, 500, 1000, 2000, 4000 and 8000 Hz to determine the hearing sensitivity levels. The air-conduction PTAs were calculated using two different formulas and rounded to the nearest whole number: pure-tone average (250, 500, 1000, 2000, 4000 and 8000 Hz) as per BSA [19] protocol and low frequency average (250 and 500 Hz). The degree of hearing loss was determined as per BSA protocol [19].
- Otoacoustic emissions (OAEs), Transient-evoked and distortion product otoacoustic emissions (TEOAEs and DPOAEs), were used to measure the functioning of the cochlear outer hair cells. Normal response was considered the finding of overall TEOAEs amplitude +12 dB or amplitude of +6 dB in at least three adjacent frequency bands.
- Auditory-evoked brainstem responses (ABRs)—ABRs are sensitive to auditory nerve and brainstem function abnormalities and thus useful in evaluating undetected damage to the auditory system [20, 21]. Monaural alternating click stimuli of 100 msec were presented at a rate of 11.1/sec via headphones. The electrical activity was amplified and filtered (range: 100–3000 Hz). A total of 1000 stimuli were given, with a mean window of 10 msec.
- Speech-in-quite test—the Arthur Boothroyd speech recognition test [22] was performed to assess speech intelligibility in quiet.

### Auditory Processing Assessment

Auditory processing disorder (APD) is defined as a specific deficit in the processing of auditory information along the central auditory nervous system, including bottom-up and top-down neural connectivity [23]. Psychoacoustic test batteries of speech and nonspeech stimuli are used to assess auditory processing in the central auditory nervous system. Temporal resolution (auditory temporal processing) is dependent on neural synchrony [24] and reported to be impaired in auditory neuropathy and FRDA patients [4–6]. Speech in noise test (auditory stream segregation) is also impaired in individuals with auditory neuropathy, FRDA [8] and brainstem pathology [25]. Thus, a clinical temporal resolution test, gaps in noise test and a speech understanding in noise test, Listening in Spatialized Noise-Sentences Test, were utilized to assess the auditory processing of the FRDA patients in our cohort.

*Gaps in Noise Test* (GIN)—GIN measures temporal resolution, which is the ability to follow rapid changes in the envelope of an auditory stimulus over time [26]. The GIN provides an estimate of threshold (shortest gap identified), a total percentage correct responses score and an estimate of attention levels.

*Listening in Spatialized Noise-Sentences Test (LiSN-S)*—the LiSN-S is a clinical speech understanding in noise test which measures the listener’s capacity to segregate a target speech signal from a competing speech noise and assesses binaural speech perception and the ability to integrate interaural timing cues in particular [27]. The test is administered under headphones, but a three-dimensional auditory environment is created by synthesizing the stimuli with head-related transfer functions [27]. Speech reception threshold (the signal-to-noise ratio required for the listener to identify 50% of the words in target sentences) is established in four conditions which vary in terms of the location of the noise source (0 versus 90 azimuth) and vocal quality of the speaker (same or different talker used to produce the target and background signals). Two “advantage” measures representing the dB benefit afforded by “spatial” or “talker” cues are also calculated. For the LiSN-S, z-scores for each of the four conditions and the spatial advantage measure were generated according to age-specific normative data [28].

### Hearing Questionnaires

The Speech, Spatial and Qualities of Hearing (SSQ) questionnaire measures a range of hearing disabilities across several hearing functions that are presumed to be served to advantage by the binaural system [29]. Bamiou et al. [30] found a correlation between the GIN test and SSQ scores indicating that temporal processing deficits may play an important role in clinical presentation. The SSQ consists of 49 questions organized into 3 sub-tests (Speech, 14 items; Spatial, 17 items; Quality, 18 items). Each item is rated from 0 (inability to hear) to 10 (perfect hearing). This questionnaire is completed in the clinic by the patient with the help of his/her communication partner as needed.

### The Montreal Cognitive Assessment (MoCA)

When referring to auditory perception, one important everyday skill is speech perception which is known to be related to cognition [31, 32]. Specifically, the performance on speech perception is linked with working memory measurements [33]. However, the accuracy of some cognitive measurements may be negatively influenced when the auditory sensory system is not functioning properly [17, 18, 34]. Research in FRDA suggests some impaired cognitive skills such as attention and working memory in these patients [35, 36], both of which are confounding factors in the central auditory processing function. In order to control for confounding cognitive factors, we collected some information on the cognitive status of patients using the Montreal Cognitive Assessment (MoCA), which is a brief screening test that assesses a wide range of cognitive functions sensitive to mild cognitive impairment. MoCA [37] that includes sections on visuospatial/executive function, naming, attention, language, abstraction, memory and orientation to time and place (30-point test cut-off of < 26/30 for mild cognitive impairment) was conducted by a qualified specialist at the Ataxia Centre at UCLH as part of routine clinical assessment.

### Statistical Analysis

Data were analysed using the Statistical Package for the Social Science (IBM SPSS Statistics for Windows, Version 22.0; IBM Corp., Armonk, NY). Differences in mean size of the GAA1 and GAA2 repeats between groups categorized on the presence of audiological abnormality (LiSN-S, normal vs. abnormal; ABR, normal vs. abnormal; GIN, normal vs. abnormal; PTA, normal vs. abnormal) were assessed for statistical significance by two-tailed *t* test. A *p* value < 0.05 was taken to indicate statistical significance. A series of hierarchical multiple linear regression analysis was used to explore associations between LiSN-S scores, GIN thresholds, subscores of SSQ questionnaire and a range of genetic variables including the repeat length of GAA1 and GAA2. The controlled factors were disease duration, MoCA and SARA scores. A partial correlation was run to determine the relationship between LiSN-S scores, GIN thresholds, subscores of SSQ questionnaire and MoCA score whilst controlling for GAA1, disease duration and SARA scores. One-way analysis of variance (ANOVA) and post hoc Bonferroni were used to compare mean GIN thresholds in patients with GAA1 less than 500, 500–700 and more than 700.

## Results

### Baseline Audiological Assessment and Association Between GAA Repeat Sizes and the Presence of Audiological Abnormalities

Hearing assessment of six FRDA patients with GAA1 repeats less than 500 revealed no/mild hearing impairment (patients 1–6). Mild/moderate hearing impairment was observed in 21 patients with GAA1 repeat lengths of more than 500 (patients 7–27). The association between the size of GAA1 and GAA2 repeats and the presence or absence of audiological abnormalities were assessed. The mean allele sizes were compared in those with audiological abnormalities and those without. A significant association with GAA1 size is observed for PTA average (*P* = 0.02), ABR (*P* = 0.000), SiQ (*P* = 0.012), LiSN-S (*P* = 0.04) and GIN (*P* = 0.000). The GAA2 was associated with the presence of abnormality in GIN test. The results are summarized in Tables 3 and 4.

**Table 3:** Distribution of audiological findings in FRDA patients

Audiological presentation	No. of patients *N* (27)	Age (years old)	GAA1	GAA2	*p* value GAA1	*p* value GAA2
PTA (BSA average)						
Normal Abnormal	15/27 (55%) 12/27 (45%)	28.9 37.5	583 810	866 1026	0.02*	0.17
PTA (LF average)						
Normal Abnormal	15/27 (55%) 12/27 (45%)	28.5 36.3	601 750	865 1015	0.14	0.14
OAEs						
Present Absent	23/27 (85%) 4/27 (15%)	31.1 42.5	661 818	922 1100	0.27	0.21
ABR						
Normal Abnormal	6/26 (23%) 20/26 (77%)	34.3 31.1	289 802	720 1015	0.000*	0.45
SiQ						
Normal Abnormal	5/22 (23%) 17/22 (77%)	32.8 31.1	380 724	796 950	0.012*	0.28
SiB						
Normal Abnormal	0/18 (0%) 18/18 (100%)	27.6	687	925	-	-
LiSN-S						
Normal/Mild SPD SPD	3/20 (15%) 17/20 (85%)	35.6 29.2	340 719	618 976	0.04*	0.06
GIN						
Normal Abnormal	7/21 (33%) 14/21 (77%)	32.3 29.6	369 722	738 1010	0.000*	0.04*

*denotes significance. LF, low frequency; BSA, British Society of Audiology; PTA, pure-tone audiometry; OAEs, otoacoustic emissions; ABR, auditory-evoked brainstem responses; SiQ, speech in quiet; SiB, speech in babble; LiSN-S, listening in spatialized noise sentences; GIN, gaps in noise test; Ab, abnormal

**Table 4:** Verbal audiological assessment and gaps in noise test details in FRDA patients

Subject	Right GIN	Left GIN	Right SiQ	Left SiQ	Right SiB	Left SiB	LiSN-S HC	LiSN-S LC	LiSN-S SA	LiSN-S TA	LiSN-S TotA	LiSN-S pattern
FRDA1*	5	5	WNL	WNL	Ab	Ab	−3.50	−1.20	−3.10	−0.40	−3.10	SPD
FRDA2*	5	6	WNL	WNL	-	-	-	-	-	-	-	-
FRDA3*	6	6	Roll over	Roll over	Ab	Ab	−2.50	−1.20	−2.20	−0.40	−3.10	Mild SPD
FRDA4*	6	6	WNL	WNL	-	-	−0.90	−1.30	−0.80	−1.20	−0.30	WNL
FRDA5*	5	5	WNL	WNL	Ab	WNL	−0.90	−0.10	−2.00	−0.50	−0.80	Mild SPD
FRDA6*	6	6	WNL	Roll over	-	-	−6.90	−5.10	−7.30	−2.50	−4.80	Mild SPD
FRDA7**	8	8	Roll over	Roll over	Ab	Ab	−2.90	−3.50	−3.10	−0.40	−1.20	SPD
FRDA8**	10	10	Roll over	Roll over	Ab	Ab	−0.80	−2.20	−3.20	−2.70	−2.90	SPD
FRDA9**	8	10	Roll over	Roll over	Ab	Ab	−3.20	−3.50	−3.40	−1.60	−1.70	SPD
FRDA10**	8	8	Roll over	Roll over	Ab	Ab	−4.40	−3.70	−3.80	−1.00	−2.80	SPD
FRDA12**	8	8	Roll over	Roll over	-	-	−2.60	−0.60	−4.30	−1.50	−2.40	SPD
FRDA14***	10	8	Roll over	Roll over	Ab	Ab	−9.00	−8.10	−7.00	−3.50	−5.60	SPD
FRDA15***	10	10	Roll over	Roll over	-	-	−7.90	−9.30	−5.40	−2.50	−3.70	SPD
FRDA16***	8	8	Roll over	Roll over	Ab	Ab	−5.40	−2.80	−4.90	−2.90	−4.30	SPD
FRDA18***	8	8	Roll over	Roll over	Ab	Ab	−3.30	−2.00	−5.80	−2.60	−2.50	SPD
FRDA20***	8	8	Roll over	Roll over	Ab	Ab	−5.80	−2.20	−4.00	−3.90	−8.10	SPD
FRDA21***	8	6	WNL	Roll over	-	-	−3.30	−2.00	−5.80	−2.50	−2.50	SPD
FRDA22***	10	10	Roll over	Roll over	Ab	Ab	−4.40	−3.70	−3.80	−1.00	−2.80	SPD
FRDA23***	10	10	Roll over	Roll over	-	-	−7.40	−6.00	−5.80	−3.20	−5.00	SPD
FRDA24***	8	10	Roll over	Roll over	Ab	Ab	−4.30	−1.20	−5.70	−2.80	−5.50	SPD
FRDA27***	10	10	Roll over	Roll over	Ab	Ab	−12.90	−16.30	−6.70	−3.10	−5.70	SPD

GIN, gaps in noise test; SiQ, speech in quiet; SiB, speech in babble; LiSN-S. listening in spatialized noise sentences; LC, low cue; HC, high cue, SA, spatial advantage; TA, talker advantage; TotA, total advantage; SPD, spatial processing disorder; Ab, abnormal; WNL, within normal limits; *, GAA1 <500; **, GAA1 500-700; ***GAA1 >700

### Binaural Speech Perception—LiSN-S Test

**Fig. 1:**
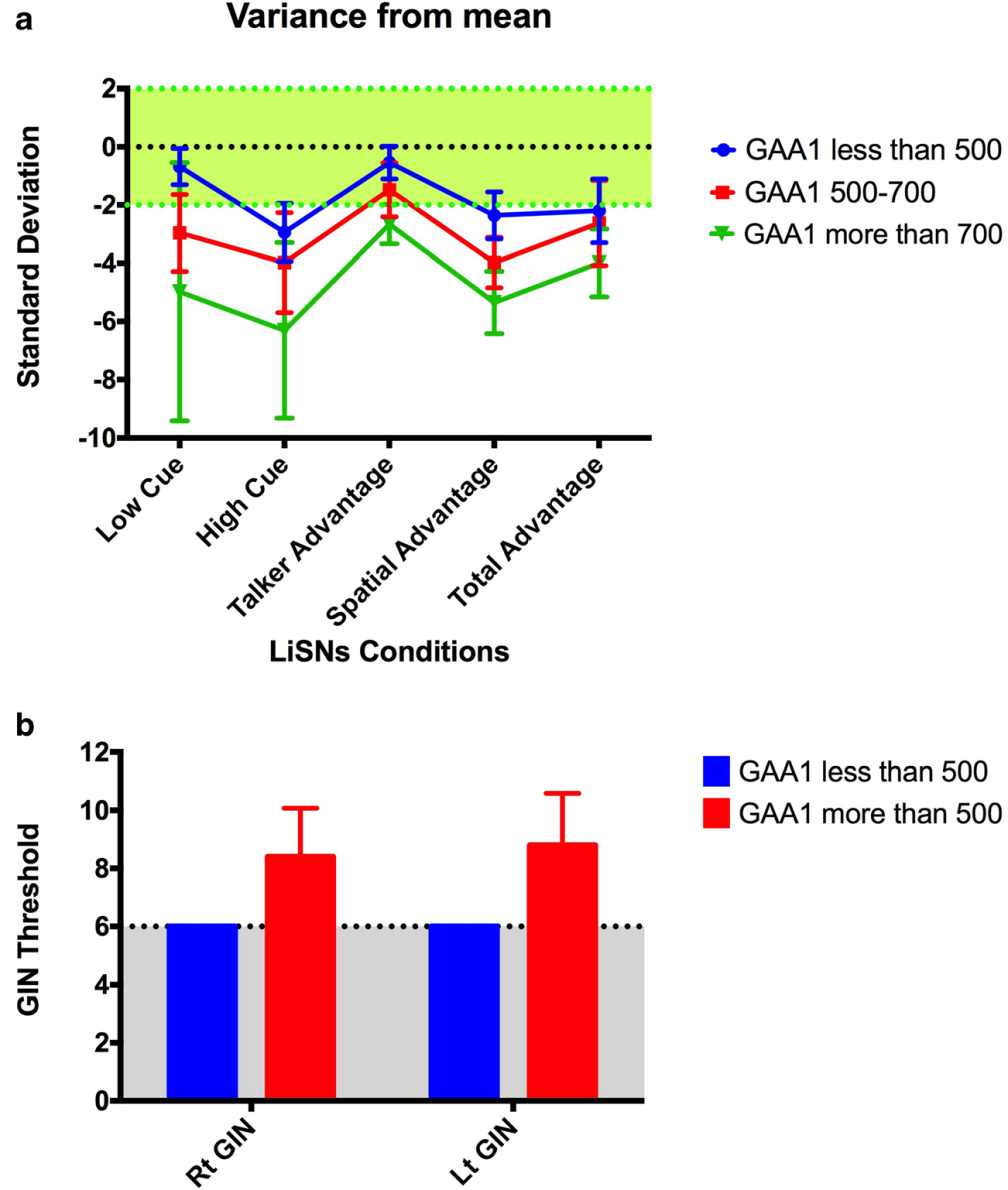
Speech reception thresholds for five subscores of LiSN-S and GIN results in FRDA patients. **a** Normal range is shaded in green. Blue line depicts the mean LiSN-S subscores for patients with GAA1 repeats less than 500; red line for GAA1 500 to 700; and green line GAA1 more than 500. **b** Normal range is below the dotted line. GIN, gaps in noise test

**Fig. 2:**
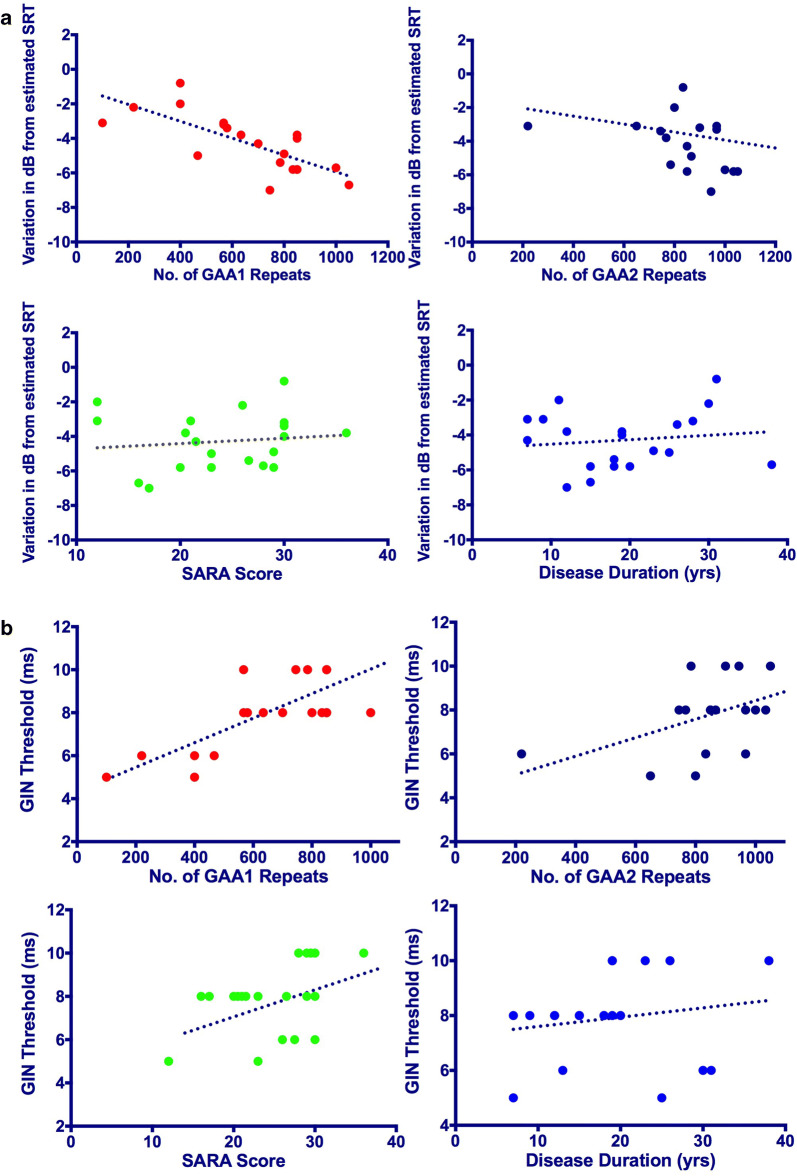
Relationship between the spatial advantage subscore of LiSN-S and GIN threshold with genetic and clinical variables. a Relationship between the spatial-advantage subscore and GAA1 repeats, GAA2 repeats, SARA score and disease duration findings for each FRDA subject. **b** Relationship between the GIN threshold and GAA1 repeats, GAA2 repeats, SARA score and disease duration findings for each FRDA subject

Summary measures (z-scores) of the five subscores of LiSN-S for the three GAA groups are shown in Fig. 1a. A series of hierarchal multiple linear regressions were calculated to predict LiSN-S scores on GAA1 and GAA2 repeats after controlling for disease duration, SARA and MoCA scores. Preliminary analyses were conducted to ensure no violation of the assumptions of normality and collinearity. No association was found between findings for two of the listening conditions (talker and total advantage) and GAA1 and GAA2 repeats size. No association was found between findings for all of the listening conditions and GAA2 repeats size. However, a hierarchal multiple linear regression analysis to predict LiSN-S spatial advantage score from GAA1 repeats showed a significant equation (F(4, 16) = 6.9, *p* < 0.05), with R squared of 0.7 and an adjusted R squared of 0.6. A significant association was also found between the LiSN-S high-cue subscore (F(4, 16) = 4.7, *p* < 0.05), with R squared of 0.6 and an adjusted R squared of 0.5, as well as low-cue subscore (F(4, 16) = 3.5, *p* < 0.05), with R squared of 0.5 and an adjusted R squared of 0.4. Figure 2a shows spatial advantage, disease duration, SARA score, GAA1 and GAA2 repeats findings for each FRDA patient.

### Temporal Resolution—Gaps in Noise

GIN results were within normal limits in patients with GAA1 less than 500 (Fig. 1b). A hierarchal multiple linear regression was calculated to predict the effect of GAA1 and GAA2 repeats on GIN thresholds when controlled for disease duration, SARA and MoCA scores. A significant equation was found (F(5, 16) = 7.5, *p* < 0.05), with R squared of 0.71 and an adjusted R squared of 0.62. Similar to spatial advantage score of LiSN-S, only GAA1 added statistically significantly to the prediction, *p* < 0.05. Figure 2b shows GIN threshold, disease duration, SARA score, GAA1 and GAA2 repeats findings for each FRDA patient.

There was a statistically significant difference between the right GIN thresholds of three GAA1 groups as determined by one-way ANOVA (F(2, 16) = 19.96, *p* < 0.001) as well as left GIN thresholds (F(2, 16) = 11.42, *p* < 0.001). A Bonferroni post hoc test revealed that the GIN threshold in patients with GAA1 less than 500 was statistically significantly lower compared to those patients with GAA1 500–700 and GAA1 more than 700. There was no statistically significant difference between those groups with GAA1 500–700 and GAA1 more than 700.

### Patient-Reported Hearing Difficulties—SSQ Questionnaire

A series of hierarchical multiple linear regressions were calculated with SSQ questionnaire subscores as the dependant variables. SARA score, disease duration and MoCA scores were entered at the stage one of the regression. The genetic variables (GAA1 and GAA2) were entered at stage two and auditory processing variables (GIN and spatial subscore of LiSN-S) at stage three. The auditory processing variables statistically significantly predicted the speech subscore, *F*(7, 13) = 6.1 , *p* < 0.05, with R squared of 0.88 and an adjusted R squared of 0.73. Interestingly, for the spatial subscore, as well as the auditory processing variables, the GAA1 added statistically significantly to the prediction, *F*(7, 13) = 4.3, *p* < 0.05, with R squared of 0.83 and an adjusted R squared of 0.64. There was no relation between the Quality and total subscores, disease duration, SARA and MoCA scores, auditory processing variables, GAA1 and GAA2 repeats, *p* > 0.05.

### Cognitive Assessment—MoCA

To explore the relationship between patients’ cognitive status and speech in noise performance, a partial correlation was run to determine the relationship between the subscores of LiSN-S test and MoCA score whilst controlling for GAA1, SARA score and disease duration. There was a strong, partial correlation between the spatial advantage subscore of LiSN-S test (−4.4 ± 1.7) and MoCA score (25.6 ± 3.5) whilst controlling for GAA1 (638.04 ± 268.37), SARA score (24.83 ± 5.75) and disease duration (19.52 ± 8.67), which was statistically significant, *r*(12) = 0.62, *N* = 17, *p* = 0.018. A partial correlation was also found between the high-cue subscore of the LiSN-S test and MoCA score, *r*(12) = 0.67, *N* = 17, *p* = 0.009.

A partial correlation was run to determine the relationship between the GIN thresholds and MoCA score whilst controlling for GAA1, SARA score and disease duration. There was no partial correlation (*r*(12) = 0.13, *N* = 17, *p* = 0.67) between the GIN thresholds (7.86 ± 1.77) and MoCA score (25.6 ± 3.5) whilst controlling for GAA1 (638.04 ± 268.37), SARA score (24.83 ± 5.75) and disease duration (19.52 ± 8.67).

No correlation was found between the SSQ subscores and MoCA scores.

## Discussion

The Friedreich’s ataxia molecular defect to some extent can explain the phenotypic variability of the disease. Previous research [11, 14] prompted us to investigate the relation between the severity of the auditory impairment, as shown on behavioural and physiological audiological assessments, and the expansion size of GAA1 repeats. We demonstrate a more extensive association between genotype and auditory phenotype in patients with FRDA than previously reported. We observed better audiometric thresholds, better ABR waveforms, better auditory temporal, spatial and speech in noise processing in patients with the repeat length of GAA1 less than 500 independent from disease duration, SARA and cognitive function (MoCA scores). Notably, a previous study provided evidence that GAA1 size is the main factor determining the severity of sensory neuropathy [14], consistent with our results. They suggested that the severity of the sensory neuropathy is genetically determined. Ragno et al. [38] described patients with minimal GAA expansion on one allele (ranging from 120 to 156 triplets) and without clinical and electrophysiological signs of sensory neuropathy.

Disrupted neural activity significantly impairs timing-related perception. This could be due to desynchronization of neural activity due to demyelination of the auditory nerve [39] or disrupted axonal transmission [40]. Previous studies have shown that the auditory neural pathway disruption in FRDA individuals is probably due to axonal degeneration [41, 42] rather than desynchronization. The findings of our study suggest that temporal distortion is mainly observed in FRDA individuals with the GAA1 more than 500. All of our five FRDA patients with GAA1 less than 500 exhibited normal GIN thresholds which was significantly lower than those with GAA1 between 500 and 700 and those more than 700 (Fig. 2b). This severity was not correlated with the repeat length of GAA2 and disease duration. This is the first study to show this relationship between the temporal distortion severity and the repeat length of GAA1 in FRDA patients. Previously, Rance et al. [4] found no relationship between the repeat length of GAA1 and severity of the impaired temporal processing. One possible reason for this discrepancy is that in that cohort, only one of the 14 FRDA patients had the GAA1 less than 500. In addition, different diagnostic tests were used for assessing the auditory processing skills.

Individuals with auditory neuropathy have difficulties integrating the timing cues arriving from the left and right auditory nerves [9]. This impairs the listener’s ability to understand speech in the presence of background noise. Rance et al. [8] demonstrated that speech perception in noise for their cohort of 23 FRDA patients was particularly affected in circumstances where binaural processing might have improved perception through spatial segregation. Spatial processing was also related to overall disease severity, as measured by the Friedreich Ataxia Rating Scale (FARS). Confirming their findings, we also observed binaural impaired speech perception in 19 out of 20 FRDA patients. However, the severity of spatial processing impairment was in varying degrees and strongly associated only with the repeat length of GAA1. Unlike Rance’s study, we did not find any relationship between any components of the LiSN-S test and overall disease severity. This disparity can be explained by the difference in their cohort of patients in that they only had one patient with the GAA1 less than 500. In addition, we used age-adjusted z-scores when calculating the LiSN-S test measures (i.e. ≤ −2.0 standard deviations from age-normalized data), whilst Rance’s study used the signal-to-noise ratio measures; thus, age effects may not have been entirely accounted for.

The structural integrity of grey matter within the cerebral and cerebellar cortices in FRDA patients was investigated in imaging study by Selvadurai et al. [43] Reduced cortical thickness and volume were observed in the prefrontal cortices, insula and temporal poles, all of which are part of the central auditory nervous system (CANS). Notably, a mild spatial processing disorder was observed even in those FRDA patients with normal ABR and those with unilateral auditory neuropathy (FRDA 3–6), which suggests their hearing impairment may also be due to CANS involvement, beyond the axonopathy in the eighth nerve and auditory brainstem, and may index other CANS problems.

### Patient-Reported Hearing Difficulties

The present study set out to determine whether the presence of auditory processing deficit is related to real-life listening difficulty and if this is also related to the repeat length of GAA1. All patients whose GAA1 was more than 500 reported gross functional deficits in a variety of complex listening situations typical of those encountered in everyday life (evident on the speech, spatial and qualities of hearing scale questionnaire). With the exception of FRDA #6 with the disease duration of 30 years and SARA score of 26 and FRDA #1 with the disease duration of 11 years and SARA score of 12 who scored low in the speech subscore of SSQ, the remaining FRDA individuals with GAA1 less than 500 did not report gross difficulties in complex listening conditions. Controlled for the disease duration, SARA and MoCA scores, only the spatial subscale of SSQ was shown to be strongly related to the repeat length of GAA1 as well as auditory processing variables. The speech subscale was only strongly related to the auditory processing variables.

There are limitations in using self-report questionnaires in that it is not clear whether the reports of the FRDA patients were in fact accurate representations of their experiences. However, our findings suggest that the spatial subscore of SSQ questionnaire may be used as a screening tool to identify those patients with auditory processing deficits.

### Cognitive Assessment—MoCA

When the encoding of sound is impaired, greater cognitive resources are required for auditory perceptual processing to the detriment of other cognitive processes such as working memory [35, 36, 44]. There is emerging research to suggest that patients with Friedrich’s Ataxia are at risk of cognitive impairment that may impact on these individuals’ communication. The findings of our study revealed a strong correlation between the MoCA score and two subscores of the LiSN-S test (spatial advantage and high-cue score). A previous study in children with auditory processing disorder [45] showed no association between the LiSN-S-derived subscores and cognitive processes such as auditory working memory and nonverbal intelligence. Long-term auditory deprivation is thought to impact cognitive performance through the reduction in communication and social activities [46]. The results of our study suggest auditory deprivation may have a role in developing mild cognitive impairment in FRDA patients. One way to delay the synergistic effect of auditory deprivation on cognitive processing is to increase the listener’s signal-to-noise ratio (SNR) [47], which acts as a bottom-up strategy. The improved SNR facilitates better auditory processing and enhanced understanding speech in noise, due to a clearer signal, which subsequently leads to reduced cognitive load and better cognitive abilities that can be used for other higher cognitive processes such as attention, memory, emotion and language processing.

### Conclusions, Limitations and Future Directions

In summary, the findings of our study suggest the presence of a more severe auditory processing disorder (APD) in those patients with a GAA1 more than 500 leading to more problems with speech perception in background noise. APD’s main symptom is difficulty understanding speech in background noise which is considered one of the most incapacitating elements of auditory impairment as it may cause feelings of isolation and affect relationships. Identifying and understanding the cause of this reported difficulty in FRDA patients is essential for hearing rehabilitation of these individuals. Additionally, the auditory processing measures may be candidate auditory biomarkers for FRDA severity and intervention benefit.

Future research should aim to test a larger cohort of FRDA patients, particularly those with GAA1 less than 500. Prospect studies should include auditory phenotyping of the paediatric population in order to characterize and classify the auditory impairments, using a detailed audiological assessment test battery. This would enable specific guides for management and intervention for this patient population.

As well as auditory processing deficits, current research suggests that FRDA patients are at risk of cognitive impairment that may also impact their ability to communicate effectively [35, 36]. The ear’s peripheral auditory systems, the central auditory pathways and cognitive systems in the brain must all work in harmony for successful communication in noisy environments [32, 48]. In particular, there is a link between speech perception performance and working memory, in which information is stored and manipulated temporarily [33], as well as verbal memory [48, 49]. Thus, as well as impaired hearing, cognitive deficits may make it more difficult to understand speech when background noise is present, with consequent worsening of the ability to communicate. Although studies have demonstrated hearing and cognitive impairment in FRDA patients, none, to the best of our knowledge, have examined both hearing and cognitive impairments and how they act together to influence cognitive, emotional and physical health. There is clearly a need to better understand the associations between the hearing and cognitive impairments in FRDA patients and correlate these results with the repeat length of GAA1. Thus, future research should evaluate the relationship and interaction between the degree of auditory impairment, overall clinical severity of the disease and cognitive function using comprehensive cognitive assessment.
